# Functional Neurological Disorder Complicated by Persecutory Delusions in a General Hospital: A Case Study

**DOI:** 10.1192/bjo.2026.11797

**Published:** 2026-06-30

**Authors:** Chisom Israel Madu, Simona Ionita, Alice Green, Gavin McKay, Mohamed Ibrahim

**Affiliations:** 1 North East London NHS Foundation Trust (NELFT), London, United Kingdom; 2 Queens Hospital Romford, London, United Kingdom

## Abstract

**Aims:**

Functional neurological disorder (FND) frequently co-occurs with psychiatric conditions, most commonly anxiety, depression, and trauma-related disorders. The coexistence of FND and psychotic illness remains less well characterised, particularly in general hospital settings. The emergence of fixed persecutory beliefs in individuals with FND presents significant diagnostic, ethical, and management challenges, especially when such beliefs interfere with essential medical care. This case illustrates the complexity of assessing risk, decision-making capacity, and the role of compulsory psychiatric treatment when physical health deterioration arises from treatment refusal rather than suicidality

**Methods:**

We describe a middle-aged man admitted to a general hospital with acute onset functional neurological symptoms, including rapidly evolving limb weakness, sensory disturbance, and functional paralysis, following significant occupational stress. During admission, he developed persistent persecutory delusions characterised by profound mistrust of healthcare professionals, beliefs of deliberate harm and surveillance, and accusations of abuse. These beliefs led to sustained refusal of nutrition, investigations, and treatment, resulting in significant weight loss, electrolyte disturbances, and functional decline. Extensive medical and neurological investigations supported a functional diagnosis, with no evidence of delirium or organic pathology to account for the psychiatric presentation. Multidisciplinary management was complicated by impaired engagement and contested use of mental health legislation.

**Results:**

This case highlights diagnostic uncertainty at the interface between FND and psychotic disorder, emphasising the importance of distinguishing illness-related beliefs from fixed delusions associated with behavioural consequences. Risk arose predominantly from malnutrition, immobility, and impaired decision-making rather than deliberate self-harm. While compulsory psychiatric treatment mitigated immediate physical health risks and resulted in partial behavioural stabilisation, it also appeared to intensify feelings of injustice and mistrust. Prolonged hospitalisation and adversarial dynamics may have reinforced persecutory beliefs, underscoring the need for early integrated neuropsychiatric formulation, consistent communication, and relational safety.

**Conclusion:**

This case demonstrates that FND may coexist with psychotic illness, with significant implications for risk assessment, capacity evaluation, and treatment planning. Clinicians should remain alert to risks arising from treatment refusal and physical deterioration in the absence of suicidality. Early multidisciplinary collaboration and proportionate use of mental health legislation are essential to minimise iatrogenic harm and support recovery in medically complex patients.

